# Prediction of episode of hemodynamic instability using an electrocardiogram based analytic: a retrospective cohort study

**DOI:** 10.1186/s12871-023-02283-x

**Published:** 2023-09-22

**Authors:** Bryce Benson, Ashwin Belle, Sooin Lee, Benjamin S. Bassin, Richard P. Medlin, Michael W. Sjoding, Kevin R. Ward

**Affiliations:** 1 Fifth Eye Inc, 110 Miller Avenue, Suite 300, Ann Arbor, MI 48104 USA; 2 Department of Emergency Medicine, University of Michigan, 1500 East Medical Center Drive, Ann Arbor, MI 48109–5301 USA; 3 Max Harry Weil Institute for Critical Care Research and Innovation, University of Michigan, NCRC 10-A103 2800 Plymouth Road, Ann Arbor, MI 48109 USA; 4 Department of Internal Medicine, Division of Pulmonary and Critical Care Medicine, University of Michigan, 1500 East Medical Center Drive, Ann Arbor, MI 48109–5642 USA

**Keywords:** Blood pressure, Critical care, Heart rate variability, Hemodynamic instability, Hemodynamic monitoring, Hypotension, Predictive analytic

## Abstract

**Background:**

Predicting the onset of hemodynamic instability before it occurs remains a sought-after goal in acute and critical care medicine. Technologies that allow for this may assist clinicians in preventing episodes of hemodynamic instability (EHI). We tested a novel noninvasive technology, the Analytic for Hemodynamic Instability-Predictive Indicator (AHI-PI), which analyzes a single lead of electrocardiogram (ECG) and extracts heart rate variability and morphologic waveform features to predict an EHI prior to its occurrence.

**Methods:**

Retrospective cohort study at a quaternary care academic health system using data from hospitalized adult patients between August 2019 and April 2020 undergoing continuous ECG monitoring with intermittent noninvasive blood pressure (NIBP) or with continuous intraarterial pressure (IAP) monitoring.

**Results:**

AHI-PI’s low and high-risk indications were compared with the presence of EHI in the future as indicated by vital signs (heart rate > 100 beats/min with a systolic blood pressure < 90 mmHg or a mean arterial blood pressure of < 70 mmHg). 4,633 patients were analyzed (3,961 undergoing NIBP monitoring, 672 with continuous IAP monitoring). 692 patients had an EHI (380 undergoing NIBP, 312 undergoing IAP). For IAP patients, the sensitivity and specificity of AHI-PI to predict EHI was 89.7% and 78.3% with a positive and negative predictive value of 33.7% and 98.4% respectively. For NIBP patients, AHI-PI had a sensitivity and specificity of 86.3% and 80.5% with a positive and negative predictive value of 11.7% and 99.5% respectively. Both groups performed with an AUC of 0.87. AHI-PI predicted EHI in both groups with a median lead time of 1.1 h (average lead time of 3.7 h for IAP group, 2.9 h for NIBP group).

**Conclusions:**

AHI-PI predicted EHIs with high sensitivity and specificity and within clinically significant time windows that may allow for intervention. Performance was similar in patients undergoing NIBP and IAP monitoring.

**Supplementary Information:**

The online version contains supplementary material available at 10.1186/s12871-023-02283-x.

## Background

Sudden, unrecognized, and delayed identification of hemodynamic deterioration of patients remains a significant challenge across all levels of in-hospital care including the intensive care unit (ICU). Failure to recognize the need for re-evaluation and escalation of care can result in unplanned ICU admissions, added length of stay, and even death [1–3]. This has prompted the development and implementation of a number of electronic medical record (EMR) deterioration indices or early warning systems (EWS) as well as rapid response teams that are designed to identify high risk patients in need of re-evaluation and to optimally intervene prior to deterioration [4–6].

While studies have demonstrated that shifts in vital signs can occur prior to adverse events and that close deliberate monitoring of vital signs may improve early detection and prompt clinical action capable of averting events such as cardiac arrest, high resolution monitoring, recording, and interpretation of vital signs is difficult even in the ICU setting [7–9]. While several EMR technologies have demonstrated promise, the infrequency of EMR input (lab values, vital signs, etc.) and the subsequent validation of that input potentially reduces the effectiveness of such approaches to identify patients early [10].

Loss of heart rate variability (HRV) has been demonstrated to reflect changes in the autonomic nervous system in the setting of many states of critical illness and injury including hemorrhage, sepsis, cardiogenic shock, respiratory failure, and others, with these changes occurring prior to overt decompensation [11–21]. However, several challenges ranging from signal acquisition and sampling rates to real-time signal processing and feature extraction have limited the approach. A single lead ECG analytic was recently developed to overcome these challenges by leveraging advanced signal processing to extract HRV and ECG morphologic features associated with hemodynamic instability [11, 22, 23]. The Analytic for Hemodynamic Instability-Predictive Indicator (AHI-PI) is designed to predict hemodynamic instability before it occurs. The analytic is an FDA cleared software as a medical device (SaMD). In this analysis, we report AHI-PI’s ability to predict a future occurrence of hemodynamic instability prior to it being identifiable by vital signs.

## Methods

This was a retrospective single-center observational cohort study conducted at a quaternary care academic health system. The study was approved by the University of Michigan’s Institutional Review Board using deidentified data (HUM00092309). The Institutional Review Board waived the need for informed consent since all data analyzed was retrospective and deidentified.

The study dataset included 4,633 consecutively hospitalized adult (≥ 18 years) patient encounters with continuous ECG monitoring between August 2019 and April 2020 across multiple levels of care including the emergency department, telemetry and step-down units, and ICUs. The University has a unique data acquisition and storage system that collects, stores, and maintains a high-resolution physiologic signal database of patients including real-time physiologic signals and waveforms such as ECG, arterial blood pressure, pulse oximetry and others.

AHI-PI utilizes streaming data from a single existing lead (II) of ECG (Mason-Likar configuration) sampled at either 120 Hz (telemetry) or 240 Hz (fixed bedside monitor) to provide information regarding the patient’s predicted future risk for clinical deterioration based on the known physiologic relationship of HRV, the autonomic nervous system, and the cardiovascular system. AHI automates the extraction and analysis of ECG patterns that reflect the compensatory burden of the autonomic nervous system including signal quality assessment and processing of extracted patterns through a pretrained classification model that embeds nonlinear HRV complexity and ECG morphologic features into a signal output [11, 23, 23]. AHI-PI builds on this output and updates every two minutes, producing one of three types of outputs: red, yellow, or green, indicating high, moderate, or low risk respectively of a future episode of hemodynamic instability. AHI-PI is based on a mathematically derived extension of past AHI outputs which incorporate time-weighted dynamic scoring that forecasts the likelihood of a future episode of hemodynamic instability.

In this analysis, an episode of hemodynamic instability (EHI) is defined as the presence of hypotension (systolic blood pressure < 90 mmHg or mean arterial pressure < 70 mmHg) combined with tachycardia (heart rate ≥ 100 bpm). This combination of blood pressure and heart rate to define EHI as it relates to inpatient adverse outcomes including mortality is supported by several widely used critical care and EWS systems including the National Early Warning Score (NEWS), the electronic Cardiac Arrest Triage (eCART), the Modified Early Warning Score (MEWS), and others [4, 24–26]. Blood pressure was either taken by intermittent noninvasive blood pressure (NIBP) monitoring and recorded in the EMR after nurse validation or with continuous intraarterial blood pressure (IAP) monitoring. For patients undergoing IAP monitoring an EHI is defined more conservatively as 10 continuous minutes or more with the above heart rate and blood pressure parameters. Requiring the simultaneous presence of both tachycardia and hypotension reflects a conservative definition of hemodynamic instability and requiring this state to be sustained for at least 10 continuous minutes indicates that the derangement of vital signs is likely to be more clinically significant [27]. For patients undergoing continuous IAP monitoring, blood pressure values and heart rate were collected at 0.5 Hz. However, patients monitored with NIBP had heart rate and blood pressure measures recorded approximately once per hour across all patients. Only ECG data was used as input for the AHI-PI algorithm, while the heart rate and blood pressure values were used only to identify the onset of EHIs. Patients were monitored for as long as they had combined ECG and blood pressure monitoring. The hospital utilizes GE Healthcare’s Carescape B850 and B650 monitoring systems (GE Heath Care, Chicago IL). NIBP measured by these systems uses automated oscillometric methodology.

Since the data was not specifically collected for this analysis and given the large dataset, no formal power analysis was performed for this study. We estimated sensitivity, specificity, and other related measures for all AHI-PI outputs across NIBP and IAP monitored patient groups. For the purposes of the classification analysis, low and moderate risk AHI-PI outputs were grouped and evaluated against high-risk outputs. To evaluate the performance of the moderate risk class individually, risk ratios of an EHI for both moderate and high-risk outputs compared to low-risk outputs were calculated. Confidence intervals are also provided for each of the performance measures based on 1000 bootstrap samples with patient level replacement. This window-level analysis compares annotation of presence or absence of EHIs within a 1-h prediction time frame against the AHI-PI scores for each window. Since AHI-PI provides a continuous output, a sliding window mechanism was utilized to incorporate each AHI-PI output into the performance analysis. The number of windows available for each patient can vary based on the duration of monitoring which is representative of the intended use of AHI-PI. The distribution of ECG monitoring and EHI durations can be found in Sect. 1 of the supplementary material.

**Fig. 1:**
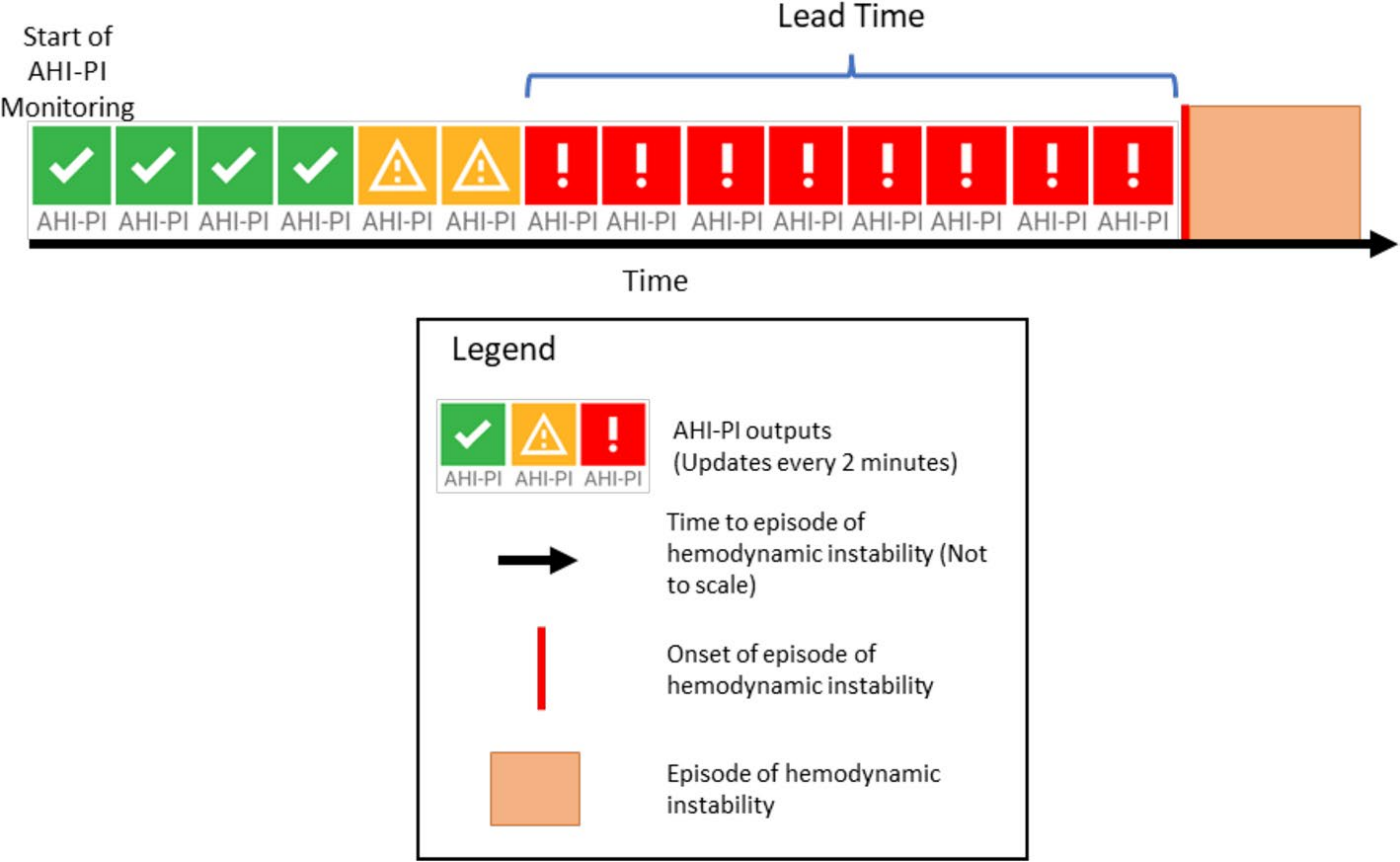
AHI-PI Lead time analysis approach. Calculation method used for AHI-PI lead time analysis

The lead time analysis assesses the question of ‘how far in advance does AHI-PI repeatedly produce a high-risk indication prior to an event?’ Therefore, using the onset of the first hemodynamic instability episode for a patient and looking back in time toward the beginning of AHI-PI monitoring, the duration of consecutive AHI-PI red (high-risk) outputs immediately prior to the onset of the EHI is calculated as the lead-time for that EHI (Fig. 1). As some patients can have more than one EHI during their hospital stay and to avoid complications of counting multiple episodes and associating those episodes with specific AHI-PI high risk indicators, only lead times associated with the onset of the first EHI during AHI-PI monitoring were considered in calculating lead-time statistics. This provides a conservative measure of AHI-PI’s ability to predict the onset of such episodes. Given the nature of this assessment, lead times were only computed on the subset of patients who experienced at least one episode of hemodynamic instability.

Data analysis was performed using MATLAB 2021a (Natick, MA) to assess the test characteristics of AHI-PI including sensitivity, specificity, positive predictive value (PPV), negative predictive value (NPV), and the resulting receiver operator areas under the curve (AUC). Differences of AHI-PI outputs between the groups were analyzed using non-parametric (Wilcoxon Rank Sum Test) as well as parametric (Student’s t-test) methods.

## Results

**Fig. 2:**
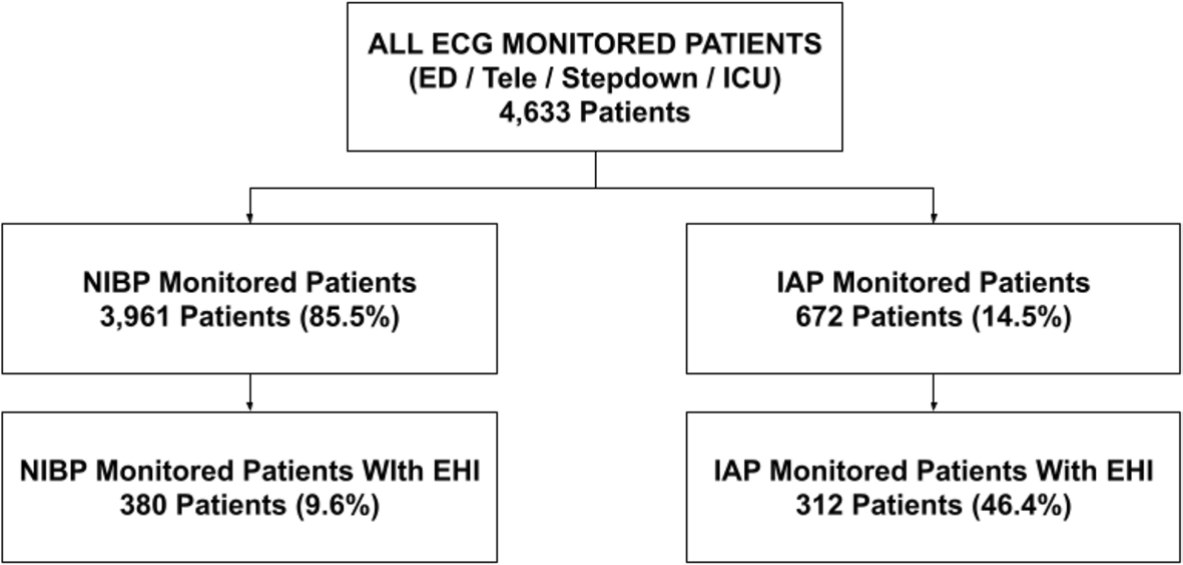
Consort diagram indicating study population and groups. NIBP: noninvasive blood pressure. IAP: intraarterial blood pressure

Figure 2 demonstrates the breakdown of captured patients and data. Of 4,633 patients, 14.5% (672) had IAP monitoring. The other 85.5% (3,961) had NIBP monitoring with blood pressure verified by nurses and imputed into the EHR. Of all IAP monitored patients, 46.4% (312) had one or more EHI, while only 9.6% (380) of the NIBP monitored patients had one or more EHI.

Table 1 provides the demographic characteristics of the study population groups. The demographic and baseline characteristics were found to be similar between each of the groups. The average age in the patient groups ranged between 60.4 to 61.4 years, with the sex distribution slightly weighted towards males. The racial and ethnic distribution in the study population reflects the distribution of patients generally seen at the University.

**Table 1:** Demographic characteristics of the study population groups

Characteristic	NIBP Monitored Patients (*N* = 3,961)	NIBP Monitored Patients With EHI (*N* = 380)	IAP Monitored Patients (*N* = 672)	IAP Monitored Patients With EHI (*N* = 312)
Sex				
Male, n (%)	2152 (54.4%)	190 (50%)	390 (58%)	182 (58.3%)
Female, n (%)	1807 (45.6%)	190 (50%)	282 (42%)	130 (41.7%)
Unknown or not reported, n (%)	2 (0.0%)	0 (0%)	0 (0%)	0 (0%)
Age (Years)				
Mean (SD)	60.4 (18.1)	61.1 (17.4)	60.6 (15.8)	61.4 (16.6)
Min, Max	18, 100	18, 99	18, 90	18, 90
Race, n (%)				
White	3188 (80.5%)	313 (82.4%)	529 (78.8%)	245 (78.5%)
Black or African American	524 (13.2%)	53 (13.9%)	91 (13.6%)	44 (14.1%)
Asian	86 (2.2%)	4 (1.0%)	14 (2.1%)	8 (2.6%)
Unknown or not reported	40 (1.0%)	5 (1.2%)	19 (2.8%)	8 (2.6%)
American Indian or Alaska Native	20 (0.5%)	1 (0.2%)	4 (0.6%)	2 (0.6%)
Native Hawaiian and Other Pacific Islander	3 (0.1%)	0 (0.0%)	1 (0.2%)	1 (0.3%)
Other	97 (2.5%)	5 (1.2%)	14 (1.9%)	4 (1.3%)
Ethnicity, n (%)				
Hispanic	114 (2.9%)	7 (1.8%)	16 (2.2%)	8 (2.6%)
Non-Hispanic	3791 (95.7%)	367 (96.6%)	635 (94.6%)	291 (93.3%)
Unknown or not reported	56 (1.4%)	6 (1.6%)	21 (3.2%)	13 (4.1%)

Table 2 provides the number and percentages of AHI-PI outputs based on low-risk, moderate-risk, and high-risk categories across both NIBP and IAP groups. Using all incidences of EHI across the three risk indicators, the risk of an EHI in the upcoming hour is 0.9% for IAP patients and 0.2% for NIBP given a low-risk AHI-PI output. Compared to this, patients demonstrating a moderate-risk output had a 6.2 (NIBP) and 6.7 (IAP) fold increased risk for an EHI in the next hour compared to a low-risk indicator. Finally, those patients having a high-risk output demonstrated a 35.7 (NIBP) and 38.9 (IAP) fold increased risk for an EHI compared to a low-risk indicator. This data formed the basis for combining the low- and moderate-risk categories to allow for performance measures.

**Table 2:** Number and percentage of AHI-PI outputs based on risk categories

AHI-PI Output Categories	Number (percentage) of Outputs
NIBP Low-risk	1,959,756 (69)
NIBP Moderate-risk	270,285 (9.52)
NIBP High-risk	610,088 (21.48)
IAP Low-risk	914,659 (61.4)
IAP Moderate-risk	139,937 (9.4)
IAP High-risk	434,770 (29.2)

AHI-PI’s sensitivity and specificity was 86.3% and 80.5% respectively for the NIBP monitored group and 89.7% and 78.3% respectively for the IAP monitored group, with an AUC of 0.87 for both groups (Table 3). PPV and NPV were 11.7% and 99.5% respectively in the NIBP group and 33.7 and 98.4% respectively in the IAP group. Since the sensitivity and specificity analysis was performed using a 1-h forward looking timeframe, AHI-PI outputs indicating high risk before the 1-h window would, in this analysis, be considered a false positive, hence negatively impacting the PPV and related measures. All data is presented in Table 3. Note that while the NIBP subset contained nearly 6 × more patients than the IAP subset, there were only 2 × the number of observations for classification-based measures. This is the result of more infrequent vital signs recordings in the NIBP population than IAP (once per hour vs 0.5 per second respectively). The low incidence rate of EHI in both groups, particularly seen in the NIBP population, naturally impacts in a negative way, the performances measures of PPV, NPV, AUPRC, and F1 score that are known to be sensitive to incidence rates [28]. Patient level aggregation of these performance measures can be found in Sect. 2 of the supplementary material.

**Table 3:** AHI-PI model performance measures in predicting presence or absence of Episodes of Hemodynamic Instability (EHI). EHI predictions within a 1-h prediction time frame against the AHI-PI scores for each window. Low and moderate risk groups are combined

**AHI-PI Window level analysis**	**NIBP Patients** [95% CI]	**IAP Patients** [95% CI]
Total Number of Patients	3,961	672
Number of AHI-PI Outputs—Low and High Risk	2,840,129	1,489,366
Incidence	2.9% [2.5%, 3.4%]	11.0% [9.7%, 12.3%]
Sensitivity	86.3% [83.8%, 88.5%]	89.7% [88.2%, 91.0%]
Specificity	80.5% [78.8%, 82.2%]	78.3% [75.7%, 80.6%]
AUC	0.87 [0.85, 0.88]	0.87 [0.86, 0.88]
Positive Predictive Value	11.7% [9.5%, 14.0%]	33.7% [30.1%, 37.3%]
Negative Predictive Value	99.5% [99.4%, 99.6%]	98.4% [98.0%, 98.7%]
False Positive Rate	19.5% [17.5%, 21.6%]	21.7% [19.0%, 24.9%]
False Negative Rate	13.7% [11.1%, 16.8%]	10.3% [8.8%, 12.1%]
AUPRC	0.13 [0.10, 0.15]	0.37 [0.33, 0.40]
F1 score	0.21 [0.18, 0.24]	0.49 [0.46, 0.52]

In the NIBP monitored patients with EHI, AHI-PI high-risk indications preceded EHI in 81.1% (308/380) of cases, with a median lead time of 1.1 h (64 min) and average lead time of 2.9 h (Table 4). Similarly in the subgroup of patients undergoing IAP monitoring (312), AHI-PI high-risk indications preceded the first EHI (10 continuous minutes of tachycardia and hypotension) in 83.3% (260/312) of cases, with a median lead time of 1.1 h (66 min) and average lead time of 3.7 h. For comparison, Table 4 also provides the distribution of the duration of ECG monitoring that was available prior to the incidences of the first EHI.

**Table 4:** AHI-PI lead time to first episode of hemodynamic instability EHR Intended Use Population. Data presented in quartiles: Q1 = 25^th^ percentile (first quartile), Q3 = 75^th^ percentile (third quartile), SD = standard deviation

Prior to First Episode of HI	ECG Monitoring duration prior to first EHI NIBP Patients	AHI-PI Prediction Lead Time NIBP Patients	ECG Monitoring duration prior to first EHI	AHI-PI Prediction Lead Time IAP Patients
Total Number of Patients	380	380	312	312
Patient with AHI-PI Lead Time	-	308, 81.1%	-	260, 83.3%
Median [Q1, Q3] Hours	6.6 [1.5, 27.2]	1.1 [0.4, 3.0]	9.1 [2.2, 32.4]	1.1 [0.4, 3.2]
Mean [SD] Hours	27.9 [51.8]	2.9 [5.2]	32.6 [70.8]	3.7 [8.4]
Min, Max Hours	0.2, 362.7	0.03, 38.9	0.2,835.1	0.03, 81.9

**Fig. 3:**
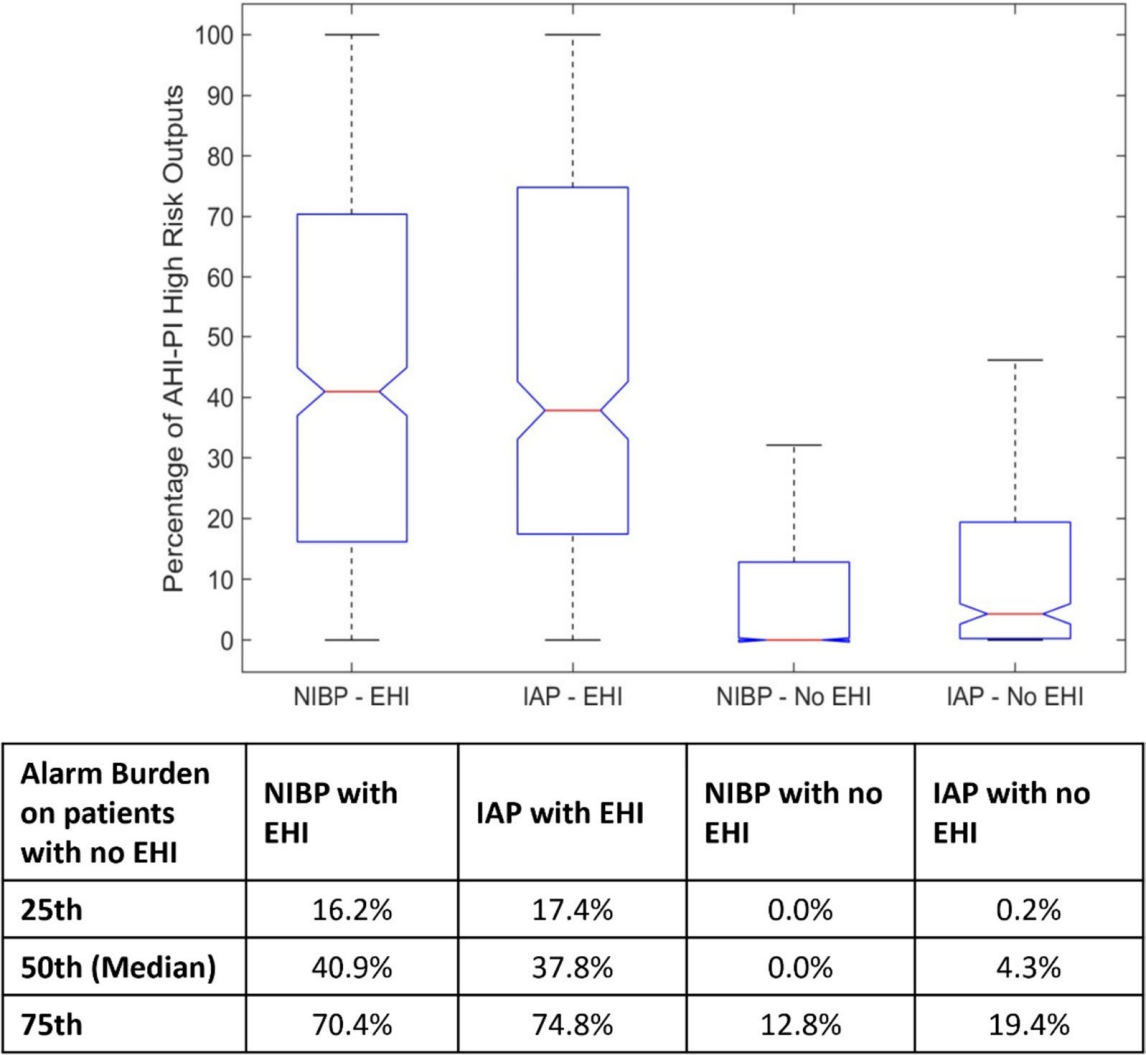
Distribution of AHI-PI High Risk indication – four groups of patients (patients with and without EHI). Patients monitored with IAP, NIBP. On each box, the central mark (red) indicates the median, and the bottom and top edges of the box indicate the 25th and 75th percentiles, respectively. The whiskers extend to the most extreme data points which are no longer than 50% greater than the interquartile range

Table 5 provides the percent of AHI-PI high-risk outputs indicated in each of the hour-long periods prior to the first EHI across ECG monitored patients with an EHI. AHI-PI demonstrated a strong indication of future risk, with 89.7% or higher of the AHI-PI outputs in each hour-long period indicating high-risk, going back to two hours from the onset of the first EHI. Additionally, the distribution of percentage of AHI-PI between patients with and without EHI within the NIBP monitored patient group (3,961) is shown in Fig. 3. The difference of AHI-PI high-risk outputs between the two groups was found to be statistically significant by both nonparametric and parametric testing with a *p*-value < 0.0001 (threshold of < 0.05 for significance). The group of patients experiencing an EHI had close to 40% of their outputs indicating AHI-PI high-risk, compared to a median of 0% in the group of patients who did not experience an EHI. This indicates that AHI-PI predominantly indicates high-risk outputs in patients who experienced an EHI.

**Table 5:** Prevalence of AHI-PI High Risk Indications in the hours prior to the onset of the first EHI across all patients (NIBP and IAP monitored)

Statistic	Monitoring Modality	0–1 h	1–2 h	2–3 h	3–4 h	4–5 h
**N patients**	NIBP	380	315	264	234	216
	IAP	312	255	220	193	171
**Median [25th, 75th]**	NIBP	100% [46.7%, 100%]	89.7% [0%, 100%]	69.1% [0%, 100%]	48.8% [0%, 100%]	45.4% [0%, 100%]
	IAP	100% [43.3%, 100%]	90.0% [0%, 100%]	61.7% [0%, 100%]	26.7% [0%, 100%]	0% [0%, 100%]
**Mean [Std Dev]**	NIBP	74.2% [38.9%]	58.8% [45.1%]	54.3% [45.8%]	48.8% [46.2%]	45.4% [45.7%]
	IAP	72.3% [39.2%]	58.5% [45.2%]	53.1% [46.1%]	45.9% [46.5%]	40.1% [46.1%]

## Discussion

In this retrospective study we examined the utility of applying the AHI-PI analytic to detect an EHI using a cohort of patients who have ECG continuously collected along with either blood pressure continuously by IAP or intermittently collected and recorded by NIBP. AHI-PI demonstrated the ability to predict an EHI with high sensitivity and specificity with lead times that can be considered clinically relevant.

Predicting acute hemodynamic deterioration or instability in a time window to allow for intervention both in the intensive care unit and general ward setting continues to present a significant challenge. While various EWSs and scores exists, including but not limited to the Modified Early Warning System (MEWS), the National Early Warning System (NEWS), the Simplified Acute Physiology Scores (SAPS), and commercial products such as the EPIC’s deterioration index (EDI), none rely on continuous real-time analysis of one or more continuously collected physiologic variables [4–6]. Instead, each are computed intermittently based on available structured data as it is imputed in the EMR. Consequently, they are contingent on the frequency of vital signs or laboratory data recorded in the EMR. As such, patients may develop physiologic changes and deterioration that occur in the interim or have causes for their hemodynamic deterioration that are not detected by the data collected from a particular EWS. Even in the ICU or operative setting, where dense high resolution physiologic signals are available, their continuous processing and analysis to predict acute adverse events such as hemodynamic instability is limited.

Other approaches and technologies have used hemodynamic waveforms to predict hemodynamic instability and hypotension. These include the Compensatory Reserve Index (CRI: Impact Vitals, Boston, MA), which uses changes in the photoplethysmograph (PPG) waveform associated with cardiac output and vasomotor changes to detect decompensation [29, 30]. The Acumen Hypotension Predictive Index system (HPI, Edwards Lifesciences, Irvine CA) uses features in the arterial waveform (collected from IAP monitoring) to predict the occurrence of hypotension during surgery [31, 32]. However, these approaches require separate hardware. AHI-PI is an SaMD that identifies HRV features associated with hemodynamic instability and is intended to be scaled to any monitor used for ECG monitoring. Its only requirement is a sampling rate of at least 120 Hz.

For AHI-PI as well as for any EWS, deterioration index, and clinical decision-making tool, there are important implications for errors. False negative outputs will mean care providers will see an AHI-PI output indicating stability when the patient is heading towards instability. If hemodynamic instability progresses unrecognized, there is the risk of failure to intervene resulting in an actual episode of instability. The high sensitivity reported in this study helps to minimize the potential to miss impending EHIs. Conversely, a false positive means the clinician will observe an AHI-PI unstable output when the patient is in fact stable and not progressing towards instability. The consequence of this can be the added resources required for increased vigilance and must be weighed in comparison to a false negative or when vital sign monitoring and reporting is less frequent. Since the AHI-PI high-risk outputs (true positives and false positives) are mainly concentrated in the group who do experience one or more EHI (Fig. 3), AHI-PI may be viewed as having low alarm fatigue implications in the group without EHI (median 0% AHI-PI high-risk Indication). Since AHI-PI is intended as adjunctive data, which may be considered by care providers in determining types of care to be initiated, continued, or discontinued, the AHI-PI is envisioned to assist in clinical decision making. Distributions of AHI-PI alerts between the two populations can be found in Sect. 3 of the supplementary material.

PPVs, AUPRC, and F1 scores between the two groups show a difference, reflecting the influence of the differences in incidence between the two groups [28]. As might be expected, the incidence of hemodynamic instability is higher in the IAP group (11.0%) as compared to the NIBP monitoring group (2.9%).

AHI was previously reported as being able to detect current hemodynamic status as opposed to predicting it [16]. The predictive aspect of AHI-PI provides a forward-looking risk assessment that supports clinical decision making. AHI-PI uses the same single lead of ECG (II) as input and combines AHI outputs that detect signs of hemodynamic instability into a single indicator that estimates the likelihood of a future adverse cardiovascular event or condition.

We believe there is a need for technology that allows for a more robust analysis of an easily obtained physiologic signal such as ECG that can be continuously analyzed to provide insight into a patient’s current and future physiologic state. We chose the ECG signal because of its ability to leverage HRV as a “vital sign” for the autonomic nervous system and its association with states of deterioration, and because of its ubiquity as a monitored signal. An additional advantage is it is much less conditioned by monitor manufacturers compared to other real-time signals and can now be monitored using a variety of adhesive patches designed for mobile monitoring. These patches allow monitoring of the ECG signal at resolutions and frequencies that provide HRV that are equivalent to traditional hospital-based monitors. The potential to use and scale such monitoring approaches may allow for more accurate triage and disposition of patients to or from higher or lower levels of care, including to general wards and even home, resulting in improved resource allocation. In addition, AHI-PI can be envisioned to guide treatments that move a patient from high-risk to low-risk status.

There are several limitations to this study. This includes the retrospective nature of the study and that the data utilized in this study comes from a single U.S. academic health center. We also used previous but well-defined definitions of stability and instability based on vital signs [24, 26, 33]. While instability characterized by only hypotension or only tachycardia can occur, we sought to use a more robust definition that combines these vital signs, which are more likely to indicate both issues with circulatory perfusion (hypotension) and the burden on the autonomic nervous systems through sympathetic activation (tachycardia), which may lead to compensatory failure and shock if left untreated [34]. Changes in these criteria would result in the need for more analysis of the performance of AHI-PI. No comparison was made of the performance of AHI-PI to other common EWS tools such SOFA, NEWS or MEWS. It will be important to understand performance differences and potential synergy with these tools in the future as well as to examine performance based on age, sex, and race. We also cannot account for potential issues or problems with misplacement of ECG leads. However, if there is non-compliance with the manufacturer recommended standard practice, the AHI-PI system has built-in signal quality assessment capabilities to automatically handle typical ECG related issues such as inverted leads, 55 Hz noise, patient motion artifacts, etc. in real-time.

It should be recognized that patients who were on a trajectory for an EHI as identified by AHI-PI may have undergone various treatments that prevented the EHI before it occurred. Patients with cardiac transplant, ventricular assist devices, sustained atrial or ventricular arrythmias, or pacemaker dependence were not excluded. These conditions could adversely impact HRV as a measure of the autonomic nervous system. Future studies that control for these issues may result in improved performances of AHI-PI. While we did not control for factors including use of vasoactive drugs or inotropes which may affect HRV we have previously demonstrated that vasopressors, inotropes, and beta blockers do not appear to significantly impact performance [23]. We also acknowledge the reported inaccuracies of noninvasive oscillometric blood pressure monitoring when compared to intra-arterial blood pressure monitoring in acute care patients [35–37]. AHI-PI in this setting may have been potentially more accurate given we paired it as a continuous measure with a significantly sparser oscillometric based NIBP measurement.

Lastly, we did not explore the reason or potential reason for patients’ EHI, what actions were undertaken to treat it, or complications from the EHI. This and the other limitations above are topics of ongoing studies.

## Conclusion

Accurate prediction of an EHI may allow for improved resource allocation, shorter time to intervene, changes in disposition, and better patient outcomes. However, such predictions are challenging when using intermittent vital signs. This study supports the potential use of a novel ECG monitoring strategy and analytic that leverages signal processing and machine learning to extract ECG features associated with impending hemodynamic instability. The noninvasive nature of the technology may offer advantages in continuous surveillance and real-time clinical decision making, facilitating interventions to prevent EHIs.

### Supplementary Information

**Additional file 1: Section 1.** Monitoring and episode duration distributions. **Section 2.** Per patient statistics. **Section 3.** Population Level Alert Distribution.

## Data Availability

The datasets generated and analyzed in this study are not publicly available due to restrictions in place by both the institutional IRB under which the study was performed as well as a data use agreement between the University and Fiftheye Inc. Limited data sharing is available from the corresponding author on reasonable request.

## Abbreviations

AHI-PI: Analytic for Hemodynamic Instability-Predictive Indicator
AUC: Area under the curve
CRI: Compensatory Reserve Index
eCART: Electronic Cardiac Arrest Triage
ECG: Electrocardiogram
EDI: EPIC’s Deterioration Index
EHI: Episode of hemodynamic instability
EMR: Electronic medical record
EWS: Early warning systems
HPI: Hypotension Predictive Index
HRV: Heart rate variability
IAP: Intra-arterial pressure
ICU: Intensive care unit
MEWS: Modified Early Warning Score
NEWS: National Early Warning Score
NIBP: Noninvasive blood pressure
NPV: Negative predictive value
PPG: Photoplethysmograph
PPV: Positive predictive value
SaMD: Software as a medical device
SAPS: Simplified Acute Physiology Scores

## References

[1] Kause J, Smith G, Prytherch D (2004). A comparison of antecedents to cardiac arrests, deaths and emergency intensive care admissions in Australia and New Zealand, and the United Kingdom–the ACADEMIA study. Resuscitation.

[2] Johnston MJ, Arora S, King D (2015). A systematic review to identify the factors that affect failure to rescue and escalation of care in surgery. Surgery.

[3] Mitchell IA, McKay H, Van Leuvan C (2010). A prospective controlled trial of the effect of a multi-faceted intervention on early recognition and intervention in deteriorating hospital patients. Resuscitation.

[4] Green M, Lander H, Snyder A (2018). Comparison of the Between the Flags calling criteria to the MEWS, NEWS and the electronic Cardiac Arrest Risk Triage (eCART) score for the identification of deteriorating ward patients. Resuscitation.

[5] Cummings BC, Ansari S, Motyka JR (2021). Predicting intensive care transfers and other unforeseen events: analytic model validation study and comparison to existing methods. JMIR Med Inform.

[6] Singh K, Valley TS, Tang S (2021). Evaluating a widely implemented proprietary deterioration index model among hospitalized patients with COVID-19. Ann Am Thorac Soc.

[7] Schein RM, Hazday N, Pena M (1990). Clinical antecedents to in-hospital cardiopulmonary arrest. Chest.

[8] Bhalala US, Bonafide CP, Coletti CM (2012). Antecedent bradycardia and in-hospital cardiopulmonary arrest mortality in telemetry-monitored patients outside the ICU. Resuscitation.

[9] Andersen LW, Kim WY, Chase M (2016). The prevalence and significance of abnormal vital signs prior to in-hospital cardiac arrest. Resuscitation.

[10] Chua WL, Mackey S, Ng EK (2013). Front line nurses' experiences with deteriorating ward patients: a qualitative study. Int Nurs Rev.

[11] Belle A, Ansari S, Spadafore M (2016). A signal processing approach for detection of hemodynamic instability before decompensation. PLoS One.

[12] Ernst G, Watne LO, Frihagen F (2017). Decreases in heart rate variability are associated with postoperative complications in hip fracture patients. PLoS One.

[13] Kleiger RE, Miller JP, Bigger JT (1987). Decreased heart rate variability and its association with increased mortality after acute myocardial infarction. Am J Cardiol.

[14] Korach M, Sharshar T, Jarrin I (2001). Cardiac variability in critically ill adults: influence of sepsis. Crit Care Med.

[15] Odemuyiwa O, Malik M, Farrell T (1991). Comparison of the predictive characteristics of heart rate variability index and left ventricular ejection fraction for all-cause mortality, arrhythmic events and sudden death after acute myocardial infarction. Am J Cardiol.

[16] Stein PK, Bosner MS, Kleiger RE (1994). Heart rate variability: a measure of cardiac autonomic tone. Am Heart J.

[17] Sztajzel J (2004). Heart rate variability: a noninvasive electrocardiographic method to measure the autonomic nervous system. Swiss Med Wkly.

[18] da Silva RB, Neves VR, Montarroyos UR (2023). Heart rate variability as a predictor of mechanical ventilation weaning outcomes. Heart Lung.

[19] Porta A, Colombo R, Marchi A (2018). Association between autonomic control indexes and mortality in subjects admitted to intensive care unit. Sci Rep.

[20] Mollura M, Lehman LH, Mark RG (2021). A novel artificial intelligence based intensive care unit monitoring system: using physiological waveforms to identify sepsis. Philos Trans A Math Phys Eng Sci.

[21] Bodenes L, N'Guyen QT, Le Mao R (2022). Early heart rate variability evaluation enables to predict ICU patients' outcome. Sci Rep.

[22] Belle A, Benson B, Salamango M (2020). A continuous real-time analytic for predicting instability in acute care rapid response team activations. Int J Med Health Res.

[23] Schmitzberger FF, Hall AE, Hughes ME (2022). Detection of hemodynamic status using an analytic based on an electrocardiogram lead waveform. Crit Care Explor.

[24] Zimmerman JE, Kramer AA, McNair DS (2006). Acute Physiology and Chronic Health Evaluation (APACHE) IV: hospital mortality assessment for today's critically ill patients. Crit Care Med.

[25] Metnitz PG, Moreno RP, Almeida E (2005). SAPS 3–From evaluation of the patient to evaluation of the intensive care unit. Part 1: Objectives, methods and cohort description. Intensive Care Med.

[26] Gardner RM, Shabot MM (2006). Patient-monitoring systems.

[27] Reich DL, Hossain S, Krol M (2005). Predictors of hypotension after induction of general anesthesia. Anesth Analg.

[28] Tenny S, Hoffman MR (2022). Prevalence.

[29] Johnson MC, Alarhayem A, Convertino V (2018). Compensatory reserve index: performance of a novel monitoring technology to identify the bleeding trauma patient. Shock.

[30] Moulton SL, Mulligan J, Grudic GZ (2013). Running on empty? The compensatory reserve index. J Trauma Acute Care Surg.

[31] Davies SJ, Vistisen ST, Jian Z (2020). Ability of an arterial waveform analysis-derived hypotension prediction index to predict future hypotensive events in surgical patients. Anesth Analg.

[32] Hatib F, Jian Z, Buddi S (2018). Machine-learning algorithm to predict hypotension based on high-fidelity arterial pressure waveform analysis. Anesthesiology.

[33] Levy MM, Fink MP, Marshall JC (2003). 2001 SCCM/ESICM/ACCP/ATS/SIS international sepsis definitions conference. Crit Care Med.

[34] Bonanno FG (2011). Clinical pathology of the shock syndromes. J Emerg Trauma Shock.

[35] Ribezzo S, Spina E, Di Bartolomeo S (2014). Noninvasive techniques for blood pressure measurement are not a reliable alternative to direct measurement: a randomized crossover trial in ICU. ScientificWorldJournal.

[36] Ilies C, Grudev G, Hedderich J (2015). Comparison of a continuous noninvasive arterial pressure device with invasive measurements in cardiovascular postsurgical intensive care patients: a prospective observational study. Eur J Anaesthesiol.

[37] Lehman LW, Saeed M, Talmor D (2013). Methods of blood pressure measurement in the ICU. Crit Care Med.

