# Supplementary material for: Prediction of episode of hemodynamic instability using an electrocardiogram based analytic: a retrospective cohort study
Source: BMC Anesthesiol. 2023 Sep 22;23:324. doi: 10.1186/s12871-023-02283-x (PMC10515416; doi:10.1186/s12871-023-02283-x)
Supplement: Supplementary file 1 — Additional file 1: Section 1. Monitoring and episode duration distributions. Section 2. Per patient statistics. Section 3. Population Level Alert Distribution. [file 12871_2023_2283_MOESM1_ESM.pdf]

Supplemental:
Additional file 1

Section 1: Monitoring and episode duration distributions

Figure 1: Distribution of ECG monitoring duration across the overall patient population. Episodes of hemodynamic instability identified across the IAP monitored patients were mostly less than 1 hour in duration (3209 of 4051 episodes).

Figure 2: Distribution of EHI durations across all patients monitored with IAP. Episodes shorter than 10 minutes were deemed ineligible and do not appear in this histogram.

Section 2: Per patient statistics

Table 1: Per patient classification results for both the IAP and NIBP populations

Section 3: Population Level Alert Distribution

Figure 3: Incidence of AHI-PI High Risk outputs in patients that exhibited at least one EHI

Figure 4: Incidence of AHI-PI High Risk outputs in patients that did not exhibit an EHI.

Section 1: Monitoring and episode duration distributions

Most commonly, patients were connected to an ECG monitor for less than 24 hours, but a subset (31) had more than 20 days of ECG monitoring. Figure 1 below shows the distribution of ECG monitoring durations across all patients in this study, both NIBP and IAP monitored.

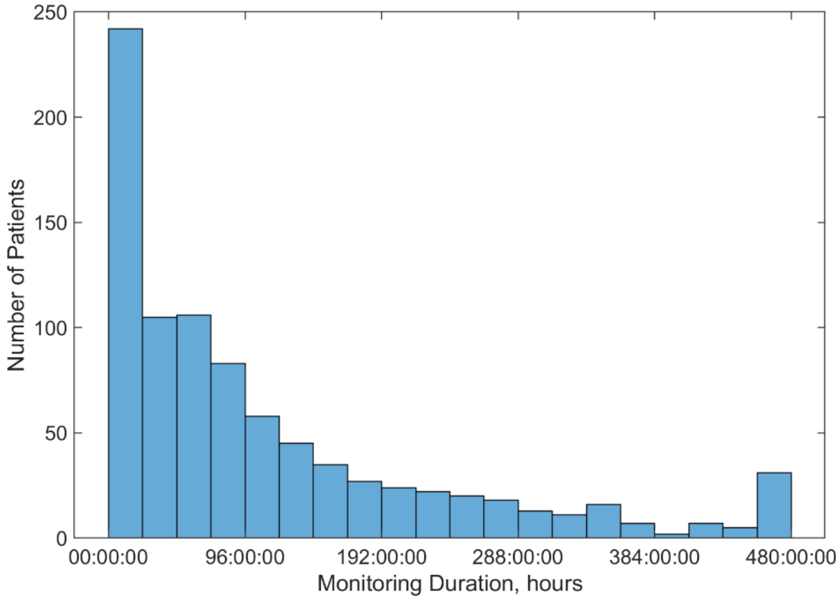

Figure 1: Distribution of ECG monitoring duration across the overall patient population.

Episodes of hemodynamic instability identified across the IAP monitored patients were mostly less than 1 hour in duration (3209 of 4051 episodes). Only hemodynamic episodes lasting at least 10 minutes were attributed to this count and multiple episodes could be counted per patient encounter. Figure 2 shows the distribution of episode durations. Note that this was only performed on the IAP patient subset due to the requirement of continuous blood pressure to assess hypotension duration.

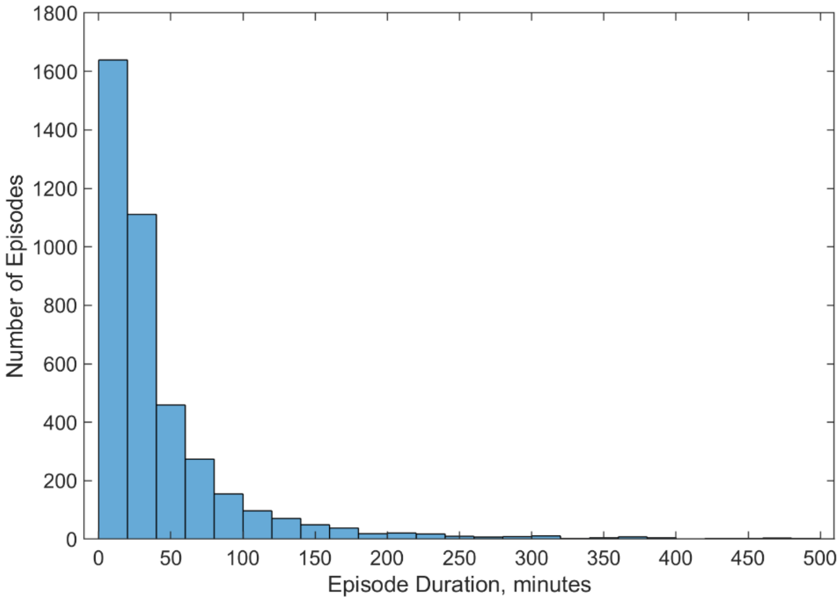

Figure 2: Distribution of EHI durations across all patients monitored with IAP. Episodes shorter than 10 minutes were deemed ineligible and do not appear in this histogram.

Section 2: Per patient statistics

Results presented in the main body of the manuscript are reported across the cohort being studied at the window level due to variability in monitoring duration and patient acuity. However, for completeness of results, the per patient results in both the IAP and NIBP populations have been presented in the table below. Results have been aggregated across patients and are presented as the median and quartiles across the populations.

Table 1: Per patient classification results for both the IAP and NIBP populations.

	**Sensitivity**	**Specificity**	**PPV**	**NPV**	**FPR**	**FNR**
**IAP**						
**25th**	80.4%	58.3%	0.0%	97.9%	2.6%	0.0%
**Median**	94.7%	88.1%	14.4%	100.0%	11.9%	5.3%
**75th**	100.0%	97.4%	49.8%	100.0%	41.7%	19.7%
**NIBP**						
**25th**	61.2%	78.6%	0%	100%	0.0%	0.0%
**Median**	94.1%	99.7%	0%	100%	0.3%	5.9%
**75th**	100.0%	100.0%	0%	100%	21.4%	38.8%

Please note that the very low incidence rate of EHI in the NIBP population impacts the patient level performance statistics for example it results in the median and quartile PPV values to be 0%.

Section 3: Population Level Alert Distribution

We are sensitive to not adding to the alarm burden. As is, the AHI-PI technology was developed to be only a visual alert and does not have a messaging alerting or alarming feature. Currently, strategies for alerting clinicians or producing audible alarms are being developed using clinicians from different unit types specific to their needs and patient acuity circumstances. The likely best practice for alarming would be to accumulate AHI-PI outputs over periods of time and trigger alarm based on specific threshold set by clinicians.

One of the key functions of the body's autonomic compensation mechanism is to continuously maintain homeostasis. A variety of conditions that affect the circulatory system, such as atherosclerosis, high blood pressure (hypertension), low blood pressure (hypotension), heart valve issues, heart failure, peripheral artery disease, and deep vein thrombosis can also affect hemodynamics and intermittently trigger the ANS compensation response. Therefore, with in-hospital patients' compensatory responses of the body can briefly and intermittently appear and vanish without it leading to overt states of hemodynamic instability. This is partly because the body self-regulates and partly because of the interventional support (fluids, pressors, etc.) these patients receive during their hospital stay. It is when the compensatory mechanism gets overwhelmed for prolonged periods of time without achieving adequate homeostasis that it can progress to EHI. Therefore, even though many patients exhibit signs of compensation which is evident through the risk indications of AHI, they might not experience EHI as defined in this study. What is important to differentiate here is the strength or proportion of the AHI-PI risk indications between patients who do ultimately experience EHI vs. those who do not. To demonstrate this, the figures below show the distribution of AHI-PI High Risk indications in patients who do not experience any EHI and patients who experience at least one EHI.

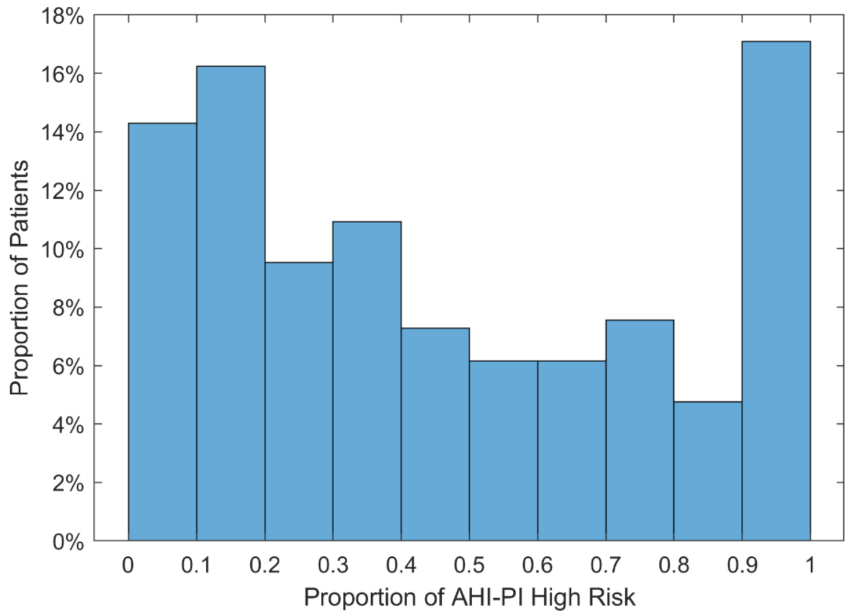

Figure 3: Incidence of AHI-PI High Risk outputs in patients that exhibited at least one EHI.

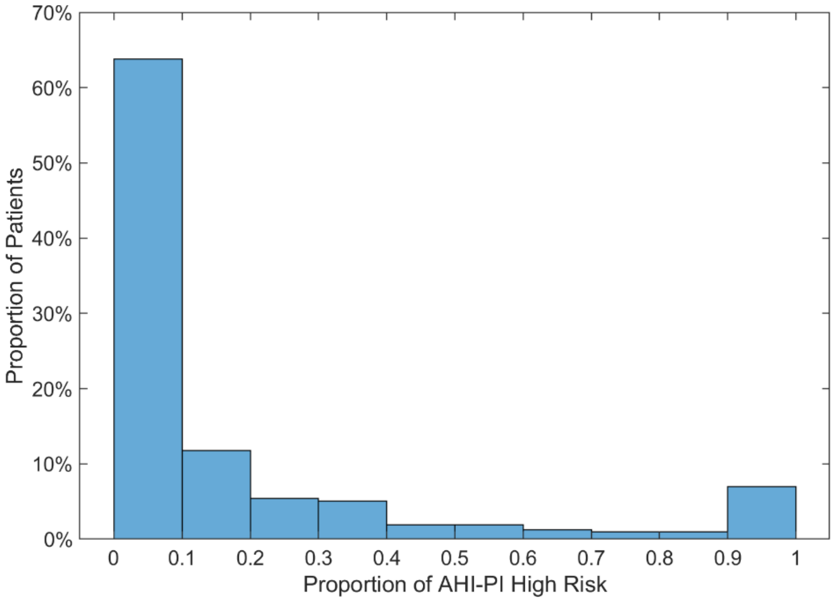

Figure 4: Incidence of AHI-PI High Risk outputs in patients that did not exhibit an EHI.
